# Concordance and Discrepancies Among 5 Creatinine-Based Equations for Assessing Estimated Glomerular Filtration Rate in Older Adults

**DOI:** 10.1001/jamanetworkopen.2023.4211

**Published:** 2023-03-23

**Authors:** Giorgi Beridze, Davide L. Vetrano, Alessandra Marengoni, Lu Dai, Juan-Jesús Carrero, Amaia Calderón-Larrañaga

**Affiliations:** 1 Aging Research Center, Department of Neurobiology, Care Sciences and Society, Karolinska Institutet and Stockholm University, Stockholm, Sweden; 2 Stockholm Gerontology Research Center, Stockholm, Sweden; 3 Department of Clinical and Experimental Sciences, University of Brescia, Brescia, Italy; 4 Department of Medical Epidemiology and Biostatistics, Karolinska Institutet, Stockholm, Sweden

## Abstract

**Importance:**

There is uncertainty as to which estimated glomerular filtration rate (eGFR) equation should be used among older adults.

**Objective:**

To compare the 5 most commonly used creatinine-based eGFR equations in older adults, quantifying the concordance among the equations, comparing their discriminative capacity in regards to 15-year mortality, and identifying sources of potential discrepancies.

**Design, Setting, and Participants:**

This cohort study used data from the Swedish National Study on Aging and Care in Kungsholmen (SNAC-K), a longitudinal study of adults aged 60 years or older in Sweden. Participants were recruited between 2001 and 2004 and followed up for mortality until December 2016. Participants missing creatinine values were excluded. Data were originally analyzed March through July 2022, and were rerun in January 2023.

**Exposures:**

Five creatinine-based equations were considered: Modification of Diet in Renal Disease (MDRD), 2009 Chronic Kidney Disease Epidemiological Collaboration (CKD-EPI), Revised Lund-Malmö (RLM), Berlin Initiative Study (BIS), and European Kidney Function Consortium (EKFC).

**Main Outcomes and Measures:**

Concordance between equations was quantified using Cohen κ. Discriminative capacity for mortality was quantified using area under the receiver operating characteristic curve (AUC) and the Harrel C statistic. Calf circumference, body mass index (BMI), and age were explored as correlates of discrepancies.

**Results:**

The study sample consisted of 3094 older adults (1972 [63.7%] female; median [IQR] age, 72 [66-81] years). Cohen κ between dyads of equations ranged from 0.42 to 0.91, with poorest concordance between MDRD and BIS, and best between RLM and EKFC. MDRD and CKD-EPI provided higher estimates of GFR compared with the other equations. The best mix of AUC and Harrel C statistic was observed for BIS (0.80 and 0.73, respectively); however, the prognostic accuracy for death decreased among those aged over 78 years and those with low calf circumference. Differences between equations were inconsistent across levels of calf circumference, BMI, and age.

**Conclusions and Relevance:**

In this cohort study, we found that eGFR equations were not interchangeable when assessing kidney function. BIS outperformed other equations in predicting mortality; however, its discriminative capacity was reduced in subgroup analyses. Clinicians should consider these discrepancies when monitoring kidney function in old age.

## Key Points

**Question:**

Can the 5 most common estimated glomerular filtration rate (eGFR) equations be used interchangeably among older adults?

**Findings:**

In this cohort study including 3094 participants, different creatinine-based equations provided divergent estimates of eGFR, with the Berlin Initiative Study equation having the highest prognostic accuracy for 15-year all-cause mortality. The differences between equations were not consistent across levels of calf circumference, body mass index, and age, suggesting that these factors may be potential sources of the observed discrepancies.

**Meaning:**

These results suggest that clinicians and researchers should carefully consider their choice of equation when monitoring kidney function in old age.

## Teaser

This cohort study of older Swedish adults examines the concordance among 5 common equations for determining estimated glomerular filtration rate for older adults and considers possible sources for discrepancies between measures.

## Introduction

Chronic kidney disease (CKD), a progressive loss of kidney function, is prevalent among 9.1% of the global population and 28% of older adults.^1,2^ It presents both as a direct cause of mortality as well as a consequence and risk factor of several diseases, and up to 2.6 million deaths worldwide in 2017 were estimated to have been attributable to CKD.^1^ Timely detection and management of kidney function deterioration can reduce progression of CKD to more severe forms, including end-stage kidney disease. However, the measurement of glomerular filtration rate (GFR), the criterion standard for assessing kidney function, is cumbersome and unfeasible for screening purposes. Indeed, obtaining measures of GFR using the clearance of exogenous filtration markers, such as iohexol, is complex and not routinely performed outside specialized settings.

The Modification of Diet in Renal Disease (MDRD) and Chronic Kidney Disease Epidemiology Collaboration (CKD-EPI) equations have been used over the last decades to obtain measures of estimated GFR (eGFR) using serum creatinine values and routinely available data such as age, sex, and ethnicity.^3,4,5^ Even if CKD-EPI remains the recommended equation by Kidney Disease: Improving Global Outcomes (KDIGO) work group for all adults,^6^ the underrepresentation of older adults in the original population (13% were older than 65 years) has yielded criticism. The transition into old age is associated with changes in body composition, including lower muscle mass and bone density as well as increased fat mass.^7^ As such, older adults with sarcopenia, a condition characterized by loss of muscle mass and function, may present with low serum creatinine even in the presence of a significant decline in GFR.^8^ In order to address this limitation, recently the Berlin Initiative Equation (BIS) was developed and specifically tailored for older adults.^9^ Other equations, such as the Revised Lund-Malmö (RLM) equation developed in Sweden, as well as the European Kidney Function Consortium (EKFC), an equation for the full-age spectrum, have also shown good accuracy across age and GFR intervals in validation cohorts.^10,11^ Several studies with different sets of equations have attempted to identify the least biased equation for older populations; however, results have been mixed and no consensus has been reached.^12^

Additionally, given the array of eGFR equations available, quantifying the concordance between equations that are commonly used in clinical practice and research is of great importance. Misclassifying individuals with CKD may delay treatment or, conversely, subject the patient to unnecessary treatment. Moreover, poor concordance among equations hinders the comparability of existing studies providing estimates of CKD prevalence or of associations between eGFR and negative health outcomes, and prevents the synthesis of the large body of information available on the topic. Several studies have reported poor concordance between the newer equations and older, traditionally used equations, and the need for further research has been highlighted.^13,14,15^

To the best of our knowledge, no study to date has quantified the concordance and explored sources of discrepancies among the aforementioned 5 eGFR equations in a large, population-based cohort of older adults with and without impaired kidney function. Therefore, we aimed to: (1) quantify the concordance among MDRD, CKD-EPI, RLM, BIS, and EKFC equations in the classification of GFR categories; (2) compare the discriminative capacity of 5 eGFR equations in regards to long-term mortality risk; and (3) explore the role of low muscle mass, low body mass index (BMI), and older age as potential sources of discrepancies among the equations.

## Methods

### Data Source and Sample Selection

This study is based on data from the Swedish National study on Aging and Care in Kungsholmen (SNAC-K),^16^ an ongoing longitudinal, community-based study of randomly sampled adults aged 60 years and above living in the Kungsholmen area of Stockholm, Sweden. Eligible participants who attended the baseline examination between 2001 and 2004 have been followed up regularly: every 6 years before the age of 78 years and every 3 years thereafter. At each study visit, participants undergo thorough clinical examinations, assessments, and interviews by physicians, trained nurses, and psychologists. Participants are also tracked in several national registers, namely the Swedish National Patient Register (specialist care medical history) and the Swedish Cause of Death Register (vital status). Out of the 3363 individuals (73.3% response rate) who attended the SNAC-K baseline examination between 2001 and 2004, 269 (8.0%) were excluded due to missing serum creatinine, resulting in a final sample of 3094 older adults.

SNAC-K was approved by the Regional Ethical Review Board in Stockholm, and written informed consent was obtained from participants or their next of kin. The study is reported in accordance with the Strengthening the Reporting of Observational Studies in Epidemiology (STROBE) guidelines for cohort studies.

### Kidney Function Assessment

Serum creatinine was measured at St. Göran’s hospital laboratory in Stockholm, where creatinine measurement was not standardized to isotope dilution mass spectrometry at the time of data collection. Thus, creatinine values were reduced by 5% before calculating eGFR.^17^ Creatinine-based eGFR was calculated using MDRD, CKD-EPI, RLM, BIS, and EKFC (full equations available in eTable 1 in Supplement 1). Participants were grouped into eGFR categories from the KDIGO guidelines^18^: G1, eGFR 90 mL/min/1.73 m^2^ or higher; G2, 89.9 to 60 mL/min/1.73 m^2^; G3a, 59.9 to 45 mL/min/1.73 m^2^; G3b, 44.9 to 30 mL/min/1.73 m^2^; and G4-5, below 30 mL/min/1.73 m^2^.

### Vital Status and Covariates

Information about the vital status of participants was available from the Swedish Cause of Death Register from start of follow-up until December 2016. Covariates included age (continuous), sex (male or female), highest attained education (elementary, high school, or university or above), BMI (continuous), and smoking (never, former, current). BMI (calculated as weight in kilograms divided by height in meters squared) was categorized as underweight (below 23), normal weight (23 to 30), and overweight (above 30) based on suggested cutoffs from a large meta-analysis conducted among older adults.^19^ Information on chronic conditions (diabetes, heart failure, cancer, hypertension) was ascertained based on clinical examinations, lab blood tests, medications, patient history, and inpatient and outpatient medical records. In short, the SNAC-K physicians combined information from all sources to create a list of *International Statistical Classification of Diseases and Related Health Problems, Tenth Revision* (*ICD-10*) diagnoses for each participant. Those *ICD-10* codes that reflect chronic health problems were later grouped into 60 broader categories of chronic conditions. Further information about the operationalization of chronic diseases in SNAC-K is available elsewhere.^20^ Calf circumference was used as a proxy for muscle mass. Low muscle mass was defined as having a calf circumference less than the 20th sex-specific percentile (below 32 cm for female and 34 cm for male individuals).^21^ Race and ethnicity were not considered due to data not being available.

### Statistical Analysis

Demographic and clinical baseline characteristics of participants were reported as median values for continuous variables and percentages for categorical variables. Cohen κ was used to quantify the concordance among different equations in the classification of GFR categories using the following cutoffs: below 0.20, poor; 0.21 to 0.40, fair; 0.41 to 0.60, moderate; 0.61 to 0.80, good; and 0.8 to 1.0, excellent.^22^ Areas under receiver operating characteristic curves (AUC) and Harrel C statistics were obtained from crude logistic regression and Cox regression models to assess how each equation predicted all-cause 15-year cumulative mortality and mortality rates. Analyses were conducted in the overall sample as well as among those with low muscle mass, those with low BMI, and those aged 78 years or older. Using the best-performing equation as reference, Bland-Altman plots were generated to plot the difference of pairs of equations against the mean of their estimates. Linear regression was used to plot the differences between the best-performing and other equations against calf circumference, BMI, and age, which were modeled using cubic splines with 5 data-driven knots. Wald tests were used to test for statistical significance. Multivariable Cox regression models were used to assess the association between eGFR (modeled using cubic splines with 5 knots prespecified at 30, 45, 60, 75, and 90) and 15-year mortality, adjusting for age, sex, education, diabetes, heart failure, cancer, hypertension, smoking, BMI, and calf circumference. Proportional hazards assumptions were tested on the basis of Schoenfeld residuals.

Statistical analysis and data visualization were performed using Stata version 17.0 (StataCorp) and Graphpad Prism 9 (GraphPad Software). Significance level was set at α < .05.

## Results

Almost two-thirds of the 3094 participants (1972 [63.7%]) were female, with a median (IQR) age of 72 (66-81) years (Table 1). Over a third (1046 [33.8%]) of the sample attained university education. Median (IQR) eGFR ranged from 61 (51-71) mL/min/1.73 m^2^ (calculated using BIS) to 69 (59-78) mL/min/1.73 m^2^ (calculated using CKD-EPI). Slightly over half (1559 participants [50.4%]) of the study population survived until the end of follow-up on December 31, 2016. Compared with the 3094 participants included in the study, the 269 excluded individuals with missing data on creatinine were more likely to be older, male, have lower education, lower calf circumference, and a higher prevalence of hypertension and heart failure (eTable 2 in Supplement 1).

**Table 1.** Baseline Characteristics of the Study Sample

Characteristic	Total, No. (%) (N = 3094)^a^
Age, median (IQR), y	72 (66-81)
Sex	
Male	1122 (36.3)
Female	1972 (63.7)
Highest attained education	
Elementary	506 (16.4)
High school	1539 (49.8)
University	1046 (33.8)
BMI	
Underweight (<23)	744 (25.4)
Normal weight (23-30)	1809 (61.8)
Overweight (>30)	374 (12.8)
Smoking status	
Never	1444 (47.0)
Former	1179 (38.4)
Current	447 (14.6)
Calf circumference, median (IQR), cm	36 (34-38)
Diabetes	276 (8.9)
Cancer	272 (8.8)
Heart failure	296 (9.6)
Hypertension	2149 (69.5)
Creatinine, median (IQR), μmol/L	82 (73-92)
eGFR, median (IQR), mL/min/1.73 m^2^	
MDRD	67 (59-75)
CKD-EPI	69 (59-78)
RLM	62 (53-70)
BIS	61 (51-71)
EKFC	63 (53-72)

Abbreviations: BIS, Berlin Initiative Study; BMI, body mass index (calculated as weight in kilograms divided by height in meters squared); CKD-EPI, Chronic Kidney Disease Epidemiological Collaboration; EKFC, European Kidney Function Consortium; MDRD, Modification of Diet in Renal Disease; RLM, Revised Lund-Malmö.

^a^ Missing data included 3 (0.1%) participants for education, 167 (5.4%) for BMI, 24 (0.8%) for smoking status, and 34 (1.1%) for calf circumference.

**Figure 1.**
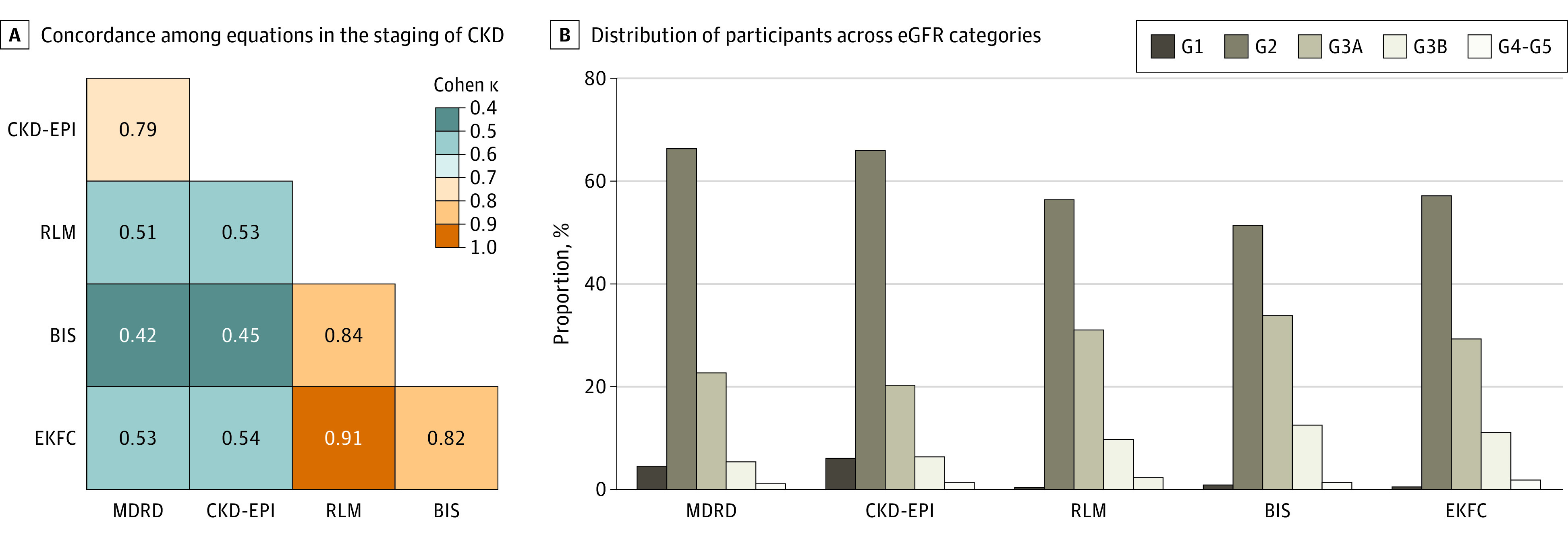
Concordance Among Equations in the Staging of Chronic Kidney Disease (CKD) and Distribution of Participants Across Estimated Glomerular Filtration Rate (eGFR) Categories G1 represents eGFR ≥90 mL/min/1.73 m^2^; G2, 89.9-60 mL/min/1.73 m^2^; G3a, 59.9-45 mL/min/1.73 m^2^; G3b, 44.9-30 mL/min/1.73 m^2^; G4-5, <30 mL/min/1.73 m^2^. BIS indicates Berlin Initiative Study; CKD-EPI, Chronic Kidney Disease Epidemiological Collaboration; EKFC, European Kidney Function Consortium; MDRD, Modification of Diet in Renal Disease; RLM, Revised Lund-Malmö.

When considering the concordance among equations with regards to classifying eGFR categories and category distribution for each equation, Cohen κ between dyads of equations ranged from 0.42 to 0.91, with the poorest concordance between MDRD-BIS and best between RLM-EKFC (Figure 1). The distribution of eGFR categories varied across equations, with MDRD and CKD-EPI placing more individuals in G1 and G2 categories compared with the other 3 equations. The prevalence of G3 and above was 29.2% using MDRD, 28.0% using CKD-EPI, 43.2% using RLM, 47.7% using BIS, and 42.3% using EKFC. The concordance between dyads including MDRD or CKD-EPI, and RLM or BIS or EKFC further decreased among the subgroups of participants aged 78 years or older and those with low muscle mass, with the poorest concordance between MDRD and BIS among those aged 78 years or older (Cohen κ = 0.17) (eFigure 1 in Supplement 1). RLM, BIS, and EKFC maintained good or excellent concordance with each other in all subgroups.

The best mix of AUC and Harrel C statistic was observed for BIS in the overall sample as well as in the subgroup analyses (Table 2). The prognostic accuracy was decreased among age 78 years or older and low muscle mass subgroups, but remained similar among those with low BMI.

**Table 2.** Discriminative Capacity of Each Equation in Regard to 15-Year All-Cause Mortality^a^

Equation	Overall (n = 3094)		Low muscle mass (n = 407)^b^		Low BMI (n = 744)^c^		Age ≥78 y (n = 1369)	
	AUC (95% CI)	Harrel C (95% CI)	AUC (95% CI)	Harrel C (95% CI)	AUC (95% CI)	Harrel C (95% CI)	AUC (95% CI)	Harrel C (95% CI)
MDRD	0.66 (0.64-0.68)	0.62 (0.61-0.64)	0.72 (0.66-0.78)	0.61 (0.58-0.65)	0.66 (0.62-0.70)	0.62 (0.60-0.65)	0.58 (0.54-0.61)	0.56 (0.55-0.58)
CKD-EPI	0.72 (0.70-0.74)	0.67 (0.66-0.69)	0.78 (0.72-0.83)	0.64 (0.61-0.68)	0.73 (0.70-0.77)	0.67 (0.65-0.70)	0.60 (0.57-0.64)	0.58 (0.56-0.60)
RLM	0.78 (0.76-0.79)	0.71 (0.70-0.73)	0.81 (0.76-0.87)	0.67 (0.64-0.70)	0.79 (0.76-0.82)	0.72 (0.69-0.74)	0.63 (0.60-0.67)	0.60 (0.58-0.62)
BIS	0.80 (0.78-0.81)	0.73 (0.72-0.74)	0.82 (0.77-0.87)	0.68 (0.64-0.71)	0.81 (0.78-0.84)	0.73 (0.71-0.75)	0.64 (0.61-0.68)	0.61 (0.59-0.62)
EKFC	0.76 (0.74-0.77)	0.70 (0.69-0.71)	0.80 (0.75-0.86)	0.66 (0.63-0.69)	0.77 (0.74-0.81)	0.70 (0.68-0.73)	0.62 (0.58-0.65)	0.59 (0.57-0.61)

Abbreviations: AUC, area under the receiver operating characteristic curve; BIS, Berlin Initiative Study; BMI, body mass index; CKD-EPI, Chronic Kidney Disease Epidemiological Collaboration; EKFC, European Kidney Function Consortium; MDRD, Modification of Diet in Renal Disease; RLM, Revised Lund-Malmö.

^a^ AUC and Harrel C statistics obtained from crude logistic regression and Cox regression models, respectively.

^b^ Low muscle mass included participants with calf circumference less than the 20th sex-specific percentile.

^c^ Low BMI included participants with BMI below 23.

Bland-Altman plots were used to compare BIS to the other 4 equations (eFigure 2 in Supplement 1). On average, MDRD and CKD-EPI provided higher estimates of eGFR by 6.0 (95% limits of agreement [LoA], −6.9 to 18.9) and 7.4 (95% LoA, −1.1 to 15.9) mL/min/1.73 m^2^ compared with BIS, respectively. Both equations showed greater differences at higher levels of eGFR. EKFC provided higher estimates of eGFR by 1.6 (95% LoA, −3.8 to 6.9) while the average bias between RLM and BIS was close to zero (0.1; 95% LoA, −4.5 to 4.8).

**Figure 2.**
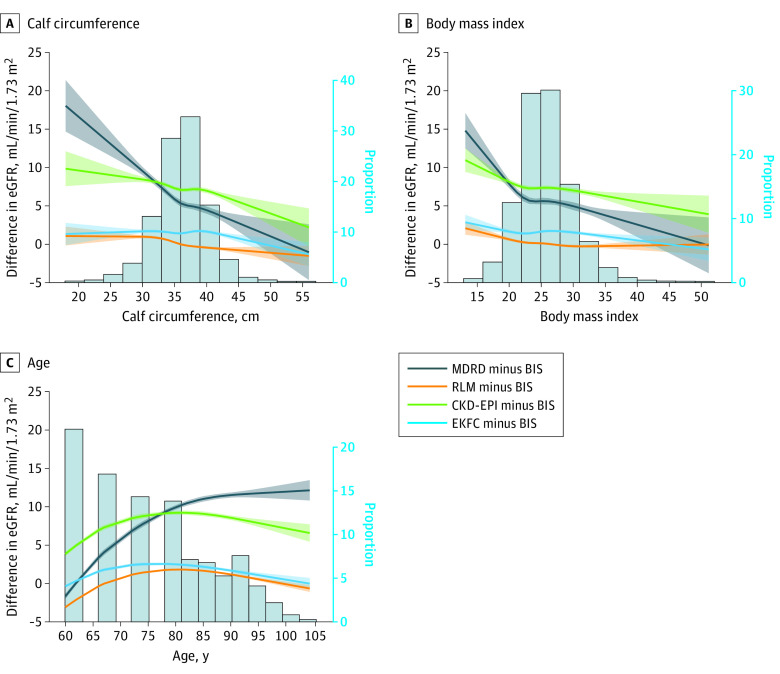
Discrepancies Between Estimated Glomerular Filtration Rate (eGFR) Equations BIS indicates Berlin Initiative Study; MDRD, Modification of Diet in Renal Disease; CKD-EPI, Chronic Kidney Disease Epidemiological Collaboration; RLM, Revised Lund-Malmö; EKFC, European Kidney Function Consortium. Shaded areas represent 95% CIs.

**Figure 3.**
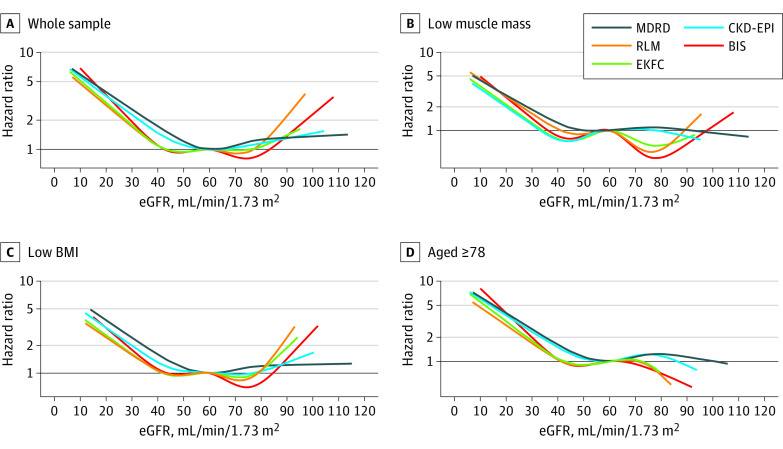
Associations Between Estimated Glomerular Filtration Rate (eGFR) Equations and 15-year Mortality All models adjusted for age, sex, education, diabetes, heart failure, cancer, hypertension, smoking, BMI, calf circumference. Low muscle mass includes individuals with calf circumference less than the 20th sex-specific percentile; low BMI, <23. BIS indicates Berlin Initiative Study; BMI, body mass index (calculated as weight in kilograms divided by height in meters squared); CKD-EPI, Chronic Kidney Disease Epidemiological Collaboration; EKFC, European Kidney Function Consortium; MDRD, Modification of Diet in Renal Disease; RLM, Revised Lund-Malmö.

Results from linear regressions using cubic splines showed that calf circumference, BMI, and age were significantly associated with the differences among all pairs of equations (all *P* < .001). The differences were more pronounced toward the lower values of calf circumference and BMI, of higher age, and of larger magnitude for MDRD and CKD-EPI compared with RLM and EKFC (Figure 2). The threshold at which the risk of mortality increases was lower for RLM, BIS, and EKFC compared with MDRD and CKD-EPI (Figure 3).

## Discussion

Our findings highlight a poor concordance among eGFR equations in this community-based cohort of Swedish older adults. Equations that were originally developed in populations where older adults were well-represented (RLM, BIS, and EKFC) showed good concordance with each other and poor concordance with equations that included fewer older adults in their development cohorts (MDRD and CKD-EPI). Both absolute differences in eGFR and concordance in CKD staging remained consistent across levels of muscle mass, body mass, and age for RLM, BIS, and EKFC. Of these equations, BIS showed the best mortality prediction, but predictive accuracy was poorer among participants aged 78 years or older and those with low muscle mass (ie, those commonly suffering from sarcopenia and underweight).

Kidney function may decline across the lifespan, and there has been a decades-long debate on how to separate age-related declines from disease-related declines. Some experts suggest that eGFR thresholds to define CKD should be age specific,^23,24^ but an age-specific definition of CKD is not yet adopted by clinical guidelines.^25^ Given that equations tend to perform best in populations similar to their development cohorts, it is not surprising that BIS, RLM, and EKFC showed better concordance with each other than with MDRD and CKD-EPI equations, and that these discordances were further amplified in subgroup analyses. Indeed, the mean age of participants was 63 in the RLM development cohort^11^ and 78.5 in the BIS cohort,^26^ while EKFC included several older adult cohorts.^10^ In contrast, older adults were far less represented in the CKD-EPI and MDRD development cohorts (mean ages of 47 and 50.6, respectively^3,27^), and the latter was developed in a cohort of CKD patients.

In addition to physiological changes in kidney function, old age is associated with changes in non–GFR-related determinants of creatinine levels. Given that the main source of serum creatinine variation is its generation after muscle breakdown, creatinine values may remain within the normal range despite a significantly impaired kidney function among individuals with low muscle mass. This could explain the large discrepancies we observed between equations at the extremes of BMI and muscle mass, as being underweight and having sarcopenia are prevalent conditions in old age that affect the metabolism of kidney function biomarkers, and equations that extrapolate findings from younger adults may not be able to fully account for such changes.

In our study, BIS outperformed all other equations in predicting mortality over the 15-year follow-up period, similar to other studies where BIS was compared with CKD-EPI.^28,29^ This was not surprising as, on the one hand, GFR is known to be associated with mortality,^30^ and on the other hand, several studies conducted in similar populations have identified the BIS equation as the least biased equation when compared with the criterion standard measured GFR (mGFR).^12^ However, even for the best-performing equation, the discriminative capacity was fair at best, particularly in subgroup analyses. This was somewhat expected given that eGFR equations were not created or validated to predict mortality, and that in our cohort of community-dwelling older adults, the prevalence of CKD is lower, and several other causes of death are competing with CKD and its complications.

The small differences observed in mortality prediction may not be as clinically relevant as the potentially large misclassification of CKD stages between different equations. In our study, CKD-EPI and MDRD, which on average provided higher estimates of GFR, had good concordance with each other but at best moderate concordance with the other 3 equations. Similar to our findings, a study conducted among 828 community-dwelling older adults aged 65 years or older in Italy found poor concordance between CKD-EPI and BIS equations (κ = 0.39) in the overall sample, and higher relative misclassification among participants aged 80 years or older and those with limitations in basic activities of daily living.^13^ Likewise, a study conducted among cardiology unit patients in Italy reported moderate concordance between MDRD and BIS, and CKD-EPI and BIS among older participants.^28^ On the other hand, a French study comparing CKD-EPI with RLM and BIS did not find significant differences between equations when comparing them to mGFR obtained by measuring iohexol clearance.^31^ The conflicting findings could be due to the differences in study populations; the French study used a clinical sample of older adults with suspected or confirmed kidney function impairment, whereas our study population included healthier participants sampled from the population. Another explanation could be that our population more closely resembled the development cohorts of the RLM and BIS equations.

Given that different CKD stages can trigger different therapeutic approaches not only in the context of CKD itself but also its comorbidities, these discordances may further complicate the health care needs encountered by older adults. Indeed, 89% of the SNAC-K population suffers from multimorbidity (defined as the presence of 2 or more chronic diseases),^20^ and at least a third of the multimorbid population is on polypharmacy.^32^ Several commonly used medications, such as methotrexate and metformin, should not be administered to those with advanced CKD as per the guidelines, and careful consideration of dosage and alternatives is needed for other antidiabetic, cardiovascular, and anticonvulsive medications with kidney clearance.^33^ Thus, diverging classifications of older peoples’ kidney function have direct and important clinical implications, and validation studies against measured GFR among the oldest patients and those with sarcopenia are highly warranted.

### Limitations

Some limitations of our study need to be highlighted. Measured GFR was not available in our study, thus we were limited to making relative comparisons between equations. We used mortality as an outcome due to its high relevance as a biological endpoint and the availability of comprehensive register data; however, the equations under study were not designed to directly assess mortality risk. We could not differentiate between transient and chronic declines in kidney function due to a single measurement of creatinine available in our study. However, this is unlikely to significantly affect the relative comparisons of eGFR equations, which was the main aim of this paper. Finally, the generalizability of our findings may be limited due to SNAC-K participants being healthier and more affluent than the average older person in Sweden. On the other hand, a major strength of the present study is that it is based on a large population-based sample of older adults with and without kidney function impairment and with comprehensive sociodemographic and clinical information available. Additional strengths include the long follow-up time and that all blood samples were collected and analyzed at a single lab in Stockholm, reducing random variability in creatinine assessment.

## Conclusions

In this population-based cohort study of Swedish older adults, we found that not all eGFR equations were interchangeable when assessing kidney function. BIS outperformed other equations with respect to mortality discrimination; however, the overall predictive capacity was only fair and further reduced in subgroups of individuals aged 78 years or older and those with low muscle mass. Validation studies against measured GFR are highly warranted in these subgroups. Until then, clinicians should consider the potential discrepancies between different eGFR equations when monitoring kidney function in old age. Accordingly, researchers should carefully consider the choice of equations in their studies.

## Notes

Supplement 1. **eTable 1.** Creatinine-based Equations Used to Calculate eGFR **eTable 2.** Characteristics of SNAC-K Participants With and Without an Available Creatinine Measurement **eFigure 1.** Concordance Among Equations in the Staging of Chronic Kidney Disease (CKD) Among Subgroups of Those With Low Muscle Mass, Low BMI and Oldest Old **eFigure 2.** Bland-Altman Plots Comparing eGFR Values Obtained Using BIS and MDRD, BIS and CKD-EPI, BIS and RLM, BIS and EKFC

Supplement 2.
Data Sharing Statement
