# Supplementary material for: Concordance and Discrepancies Among 5 Creatinine-Based Equations for Assessing Estimated Glomerular Filtration Rate in Older Adults
Source: JAMA Netw Open. 2023 Mar 23;6(3):e234211. doi: 10.1001/jamanetworkopen.2023.4211 (PMC10037147; doi:10.1001/jamanetworkopen.2023.4211)
Supplement: Supplement 1. — eTable 1. Creatinine-based Equations Used to Calculate eGFR eTable 2. Characteristics of SNAC-K Participants With and Without an Available Creatinine Measurement eFigure 1. Concordance Among Equations in the Staging of Chronic Kidney Disease (CKD) Among Subgroups of Those With Low Muscle Mass, Low BMI and Oldest Old eFigure 2. Bland-Altman Plots Comparing eGFR Values Obtained Using BIS and MDRD, BIS and CKD-EPI, BIS and RLM, BIS and EKFC [file jamanetwopen-e234211-s001.pdf]

Supplemental Online Content

Beridze G, Vetrano DL, Marengoni A, Dai L, Carrero JJ, Calderón-Larrañaga A. Concordance and discrepancies among 5 creatinine-based equations for assessing estimated glomerular filtration rate in older adults. *JAMA Netw Open.* 2023;6(3):e234211. doi:10.1001/jamanetworkopen.2023.4211

eTable 1. Creatinine-based Equations Used to Calculate eGFR

eTable 2. Characteristics of SNAC-K Participants With and Without an Available Creatinine Measurement

eFigure 1. Concordance Among Equations in the Staging of Chronic Kidney Disease (CKD) Among Subgroups of Those With Low Muscle Mass, Low BMI and Oldest Old

eFigure 2. Bland-Altman Plots Comparing eGFR Values Obtained Using BIS and MDRD, BIS and CKD-EPI, BIS and RLM, BIS and EKFC

This supplemental material has been provided by the authors to give readers additional information about their work.

© 2023 Beridze G et al. *JAMA Network Open.*

eTable 1. Creatinine-based equations used to calculate eGFR.

Name		Equation
MDRD		$175 \times SCr^{-1.154} \times age^{-0.203} \times 1.212$ (if patient is black) $\times 0.742$ (if female)
CKD-EPI (2009)	Men (Scr ≤ 0.9)	$141 \times (Scr/0.9)^{-0.411} \times 0.993^{Age}$
	Men (SCr >0.9)	$141 \times (SCr/0.9)^{-1.209} \times 0.993^{Age}$
	Women (SCr ≤0.7)	$144 \times (SCr/0.7)^{-0.329} \times 0.993^{Age}$
	Women (SCr >0.7)	$144 \times (SCr/0.7)^{-1.209} \times 0.993^{Age}$
RLM		$e^{X-0.0158\times Age+0.438\times \ln(Age)}$
	Men (SCr < 2.04)	$x = 2.56+0.00968 \times (180-SCr)$
	Men (SCr ≥ 2.04)	$x = 2.56-0.926 \times \ln(SCr/180)$
	Women (SCr < 1.7)	$x = 2.50+0.0121 \times (150-SCr)$
	Women (SCr ≥ 1.7)	$x = 2.50-0.926 \times \ln(SCr/150)$
BIS		$3736 \times SCr^{-0.87} \times age^{-0.95} \times 0.82$ (if female)
EKFC	Men (SCr/0.9 <1)	$107.3 \times (SCr/0.9)^{-0.322} \times 0.990^{(Age-40)}$
	Men (SCr/0.9 ≥1)	$107.3 \times (SCr/0.9)^{-1.132} \times 0.990^{(Age-40)}$
	Women (SCr/0.7 <1)	$107.3 \times (SCr/0.7)^{-0.322} \times 0.990^{(Age-40)}$
	Women (SCr/0.7 ≥1)	$107.3 \times (SCr/0.7)^{-1.132} \times 0.990^{(Age-40)}$

Abbreviations. MDRD: Modification of Renal Disease; CKD-EPI: Chronic Kidney Disease Epidemiological Collaboration; RLM: Revised Lund-Malmo; BIS: Berlin Initiative Study; EKFC: European Kidney Function Consortium; SCr: Serum Creatinine

© 2023 Beridze G et al. *JAMA Network Open.*

eTable 2. Characteristics of SNAC-K participants with and without an available creatinine measurement.

Characteristic	Not missing creatinine N=3,094	Missing creatinine N=269	p-value
	**No. (%)**	**No. (%)**	
**Age, median (IQR)**	72 (66-81)	88 (78-94)	<.001
**Sex**			<.001
Men	1,122 (36.3)	59 (21.9)	
Women	1,972 (63.7)	210 (78.1)	
**Education**			<.001
Elementary	506 (16.4)	84 (35.0)	
High school	1,539 (49.8)	112 (46.7)	
University	1,046 (33.8)	44 (18.3)	
**BMI**	25.2 (23.0-27.8)	24.8 (20.9-27.8)	.05
Underweight	744 (25.4)	41 (35.3)	
Normal weight	1,809 (61.8)	60 (51.7)	
Overweight	374 (12.8)	15 (12.9)	
**Smoking Status**			.02
Never	1,444 (47.0)	109 (57.1)	
Former	1,179 (38.4)	64 (33.5)	
Current	447 (14.6)	18 (9.4)	
**Diabetes**	276 (8.9)	20 (7.4)	.41
**Cancer**	272 (8.8)	27 (10.0)	.49
**Heart failure**	296 (9.6)	57 (21.2)	<.001
**Hypertension**	2,149 (69.5)	128 (47.6)	<.001
**Calf circumference (cm), median (IQR)**	36 (34-38)	32 (29-36)	<.001

Abbreviations. BMI: Body Mass Index

© 2023 Beridze G et al. *JAMA Network Open.*

eFigure 1. Concordance among equations in the staging of chronic kidney disease (CKD) among subgroups of those with low muscle mass, low BMI and oldest old.

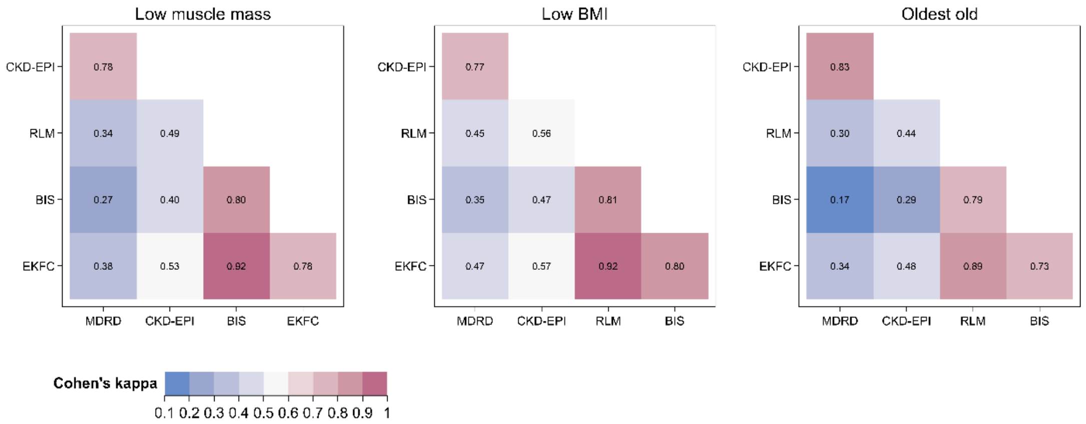

Low muscle mass: calf circumference less than the 20th sex-specific percentile; Low BMI: <23 kg/m²; Oldest old: 78 years or older.

Abbreviations. Body mass index: BMI; MDRD: Modification of Renal Disease; CKD-EPI: Chronic Kidney Disease Epidemiological Collaboration; RLM: Revised Lund-Malmo; BIS: Berlin Initiative Study; EKFC: European Kidney Function Consortium

© 2023 Beridze G et al. *JAMA Network Open.*

eFigure 2. Bland-Altman plots comparing eGFR values obtained using BIS and MDRD (panel A), BIS and CKD-EPI (panel B), BIS and RLM (panel C), BIS and EKFC (panel D). The dotted horizontal lines represent 95% limits of agreement.

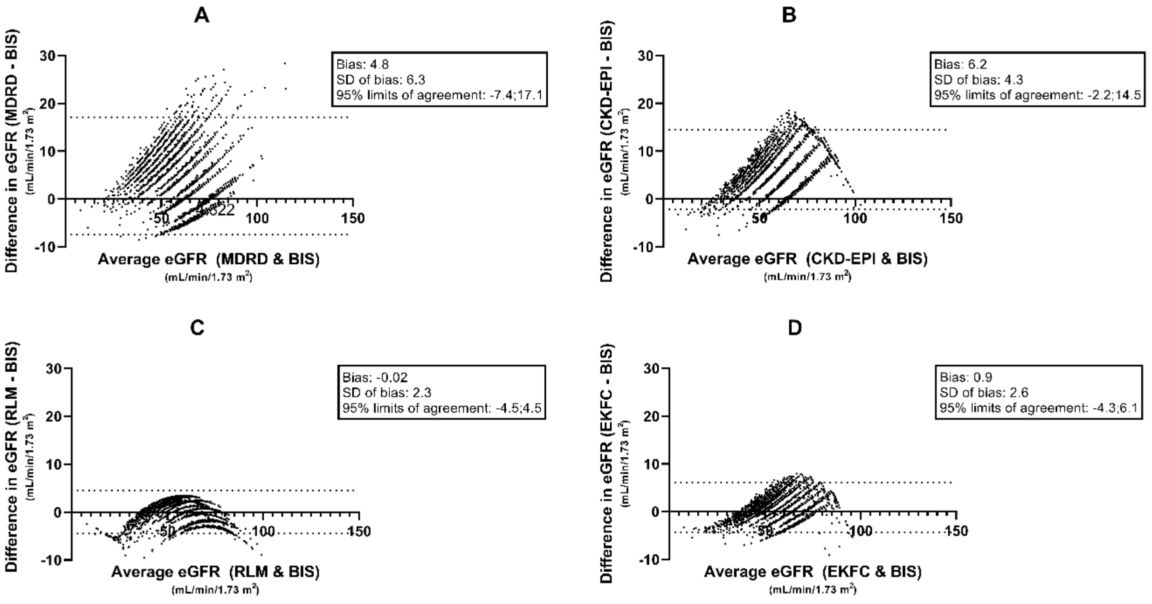

Abbreviations. MDRD: Modification of Renal Disease; CKD-EPI: Chronic Kidney Disease Epidemiological Collaboration; RLM: Revised Lund-Malmo; BIS: Berlin Initiative Study; EKFC: European Kidney Function Consortium

© 2023 Beridze G et al. *JAMA Network Open.*
