# Supplementary material for: Concordance and Discrepancies Among 5 Creatinine-Based Equations for Assessing Estimated Glomerular Filtration Rate in Older Adults
Source: JAMA Netw Open. 2023 Mar 23;6(3):e234211. doi: 10.1001/jamanetworkopen.2023.4211 (PMC10037147; doi:10.1001/jamanetworkopen.2023.4211)
Supplement: Supplement 2. — Data Sharing Statement [file jamanetwopen-e234211-s002.pdf]

Data Sharing Statement

Beridze. Concordance and Discrepancies Among 5 Creatinine-Based Equations for Assessing Estimated Glomerular Filtration Rate in Older Adults. *JAMA Netw Open*. Published March 23, 2023. doi:10.1001/jamanetworkopen.2023.4211

Data
Data available: Yes
Data types: Other (please specify)
Additional Information: Data are from the SNAC-K Project, a population-based study on ageing and dementia (http://www.snac-k.se/). Access to these original data is available to the research community upon approval by the SNAC-K data management and maintenance committee.
How to access data: Applications for accessing these data can be submitted to Maria Wahlberg (maria.wahlberg@ki.se) at the Aging Research Center, Karolinska Institutet.
When available: With publication

Supporting Documents
Document types: None

Additional Information
Who can access the data: Anyone requesting the data
Types of analyses: Case-by-case
Mechanisms of data availability: Case-by-case
